# Formulation and Characterization of Eplerenone Nanoemulsion Liquisolids, An Oral Delivery System with Higher Release Rate and Improved Bioavailability

**DOI:** 10.3390/pharmaceutics11010040

**Published:** 2019-01-18

**Authors:** Ahmed Khames

**Affiliations:** 1 Department of pharmaceutics and industrial pharmacy, Beni-suef University, Beni-Suef 62514, Egypt; dr.akhamies@Gmail.com; 2 Department of pharmaceutics and industrial pharmacy, Taif University, Taif 21944, Saudi Arabia

**Keywords:** eplerenone, nanoemulsion, liquisolids, Triacetin, bioavailability

## Abstract

Because Eplerenone (EPL) is a Biopharmaceutical Classification System (BCS) class-II drug and is prone to extensive liver degradation, it suffers from poor bioavailability after oral administration. This work aimed to prepare liquisolids loaded with EPL-nanoemulsions (EPL-NEs) that have a higher drug release rate and improved bioavailability by the oral route. Based on solubility studies, mixtures of Triacetin (oil) and Kolliphor EL/PEG 400 surfactant/co-surfactant (S_mix_) in different ratios were used to prepare EPL-NE systems, which were characterized and optimized for droplet size, zeta potential, polydispersity index (PDI), and drug content. Systems were then loaded onto liquisolid formulations and fully evaluated. A liquisolid formulation with better drug release and tableting properties was selected and compared to EPL-NEs and conventional EPL oral tablets in solid-state characterization studies and bioavailability studies in rabbits. Only five NEs prepared at 1:3, 1:2, and 3:1 S_mix_ met the specified optimization criteria. The drug release rate from liquisolids was significantly increased (90% within 45 minutes). EPL-NE also showed significantly improved drug release but with a sustained pattern for four hours. Liquisolid bioavailability reached 2.1 and 1.2 relative to conventional tablets and EPL-NE. This suggests that the EPL-NE liquisolid is a promising oral delivery system with a higher drug release rate, enhanced absorption, decreased liver degradation, and improved bioavailability.

## 1. Introduction

Eplerenone (EPL), a member of the spironolactone group, is a new selective steroidal anti-mineralocorticoid receptor blocker. EPL selectively prevents aldosterone receptor binding in both epithelial and non-epithelial tissues that results in the blockage of the renin-angiotensin-aldosterone-system (RAAS), with subsequent inhibition of sodium reabsorption that affects blood pressure and the cardiovascular system [1,2,3]. The resultant extended increase of plasma renin and serum aldosterone also inhibits the negative regulatory feedback of aldosterone on renin release [4,5]. EPL is used to treat hypertension, central serous retinopathy, and chronic heart failure either alone or in combination with other antihypertensive agents [6,7,8]. EPL is an aldosterone analogous with efficient renal and cardiac protective abilities, which is why EPL is used by elderly patients taking Angiotensin-Converting Enzyme Inhibitors (ACEIs) or Angiotensin II Receptor Blockers (ARBs) [9]. EPL also helps to reduce the acute myocardial infarction death rate in patients with systolic dysfunction of the left ventricle and heart failure [10,11].

EPL (molecular weight of 414.49 g/mol) is a crystalline powder that is slightly soluble in water (<1 mg/mL). It has a high octanol/water partition coefficient (log Kow = 7.1 at pH 7.0). EPL is a BCS Class II drug (low solubility and high permeability) of low bioavailability that is significantly affected by drug solubility [12].

After oral administration and once dissolved, EPL is rapidly absorbed from the upper gastrointestinal tract (GIT) with a negligible food effect and reaches its maximum plasma concentration within 1.5–2 hours. EPL is extensively metabolized in the liver (less than 5% of a dose is excreted in urine in an unmodified form) via CYP3A4 to inactive metabolites [1,13]. The hepatic first-pass metabolism of EPL is 12.6% and 27.1% under fasted and fed conditions, respectively, with a significant effect on drug bioavailability (69% for a 100 mg oral tablet). EPL is mainly excreted in urine with a t_1/2_ of 3 to 6 hours [14].

For efficient absorption from the GIT, EPL must reach the absorption site in a soluble form; thus, poor solubility is the main factor that retards drug absorption, decreases bioavailability, and may increase the probability of drug damage in the GIT [15]. Improving drug solubility is a major concern of drug formulators, especially for drugs classified as BCS class II drugs in which the dissolution rate represents the rate determining step of the absorption process [16,17]. Several techniques have been studied and used to increase the solubility of lipophilic drugs [16,18].

Lipid-based drug delivery systems, especially nano-sized systems including nano-lipid carriers (NLCs), solid lipid nanoparticles (SLNs), self-emulsifying drug delivery systems (SEDDSs), and nanoemulsions (NEs). Since nano-sized systems can easily mix with GIT fluids to increase drug solubility that results in enhanced absorption and improved bioavailability, they have received extensive consideration for oral delivery of poorly soluble drugs [19]. Many studies have claimed that lipid formulations escape the dissolution step that is necessary for conventional solid or suspension oral dosage forms and pass through GIT membranes via the lymphatic system, which keeps the drug away from enzymatic degradation that occurs in the hepatic first-pass effect [20,21]. This is a unique advantage for BCS class II drugs, especially those that are susceptible to enzymatic liver degradation, which significantly improves their absorption and therefore their bioavailability. 

NEs are lipid-based colloidal systems that are transparent and thermodynamically stable, with a droplet size in the range of 50–200 nm. NEs are stabilized by a surfactant/co-surfactant interfacial film that results in ultra-low interfacial tensions and high kinetic stability that prevents droplet coalescence and phase separation. In addition to high physical stability, other advantages of NEs include high solubilization ability, efficient membrane permeability, superior protection of drugs against oxidation, hydrolytic and physiological enzymatic degradation, controlled release, drug targeting, and increased bioavailability. Ease of preparation with a high cost benefit increases the potential use of NEs in pharmaceutical applications [22,23].

The liquisolid mixture has a dry appearance and is a non-adherent and flowable powder mixture containing liquid medication that is suitable for direct compression [24]. A liquisolid mixture is prepared by simple blending of a liquid medication with selected powder excipients referred to as the carrier and coating materials. Various grades of cellulose, starch, and lactose may be used as the carrier, while very fine silica powder is usually used as a coating material. The term “liquid medication” refers to drug solutions, drug suspensions, emulsions, or liquid oily drugs. Quantities of carrier and coating materials needed to produce adequately flowing and compressible properties are calculated according to a liquisolid formation mathematical model [25]. According to this model, the carrier and coating materials can retain only certain amounts of liquid (maximum liquid load) while keeping acceptable flow and compression properties, which is called the liquid load factor (L_f_). Depending on the selected excipient ratio (R) and the calculated liquid load factors, the required amounts of carrier and coating materials can be calculated [26].

A previous study [15] indicated that solubility enhancement of BCS class II drugs has a positive effect of on bioavailability at the main absorption site. The present study aimed to prepare EPL-NE loaded liquisolid tablet mixtures that combined the advantages of the NE system (i.e., higher drug absorption due to the surfactant effect on GIT permeability [27] and protection from liver degradation via para-cellular penetration [28]) with the advantages of liquisolids (i.e., rapid, high drug release at the main absorption site and presentation of NE formulations in a free flowing, highly compressible dry form suitable for oral delivery with enhanced drug bioavailability). Based on drug solubility studies, Triacetin, Kolliphor EL, and PEG 400 as an oil, surfactant, and co-surfactant, respectively were selected for preparing NE systems by the titration method. Pseudo-ternary phase diagrams were constructed to determine the area of clarity (NE area) and different plain NE systems were prepared using different S_mix_ ratios. Based on droplet size, zeta potential, and polydispersity index (PDI), NE systems were optimized for drug loading and were incorporated into liquisolid formulation prepared using Avicel/nano-silica as a carrier/coat mixture according to the proposed mathematical model. EPL-NE liquisolids were characterized before and after compression and drug release was compared to that of an unformulated EPL. An EPL-NE liquisolid formulation with higher drug release and better tableting properties was selected and subjected to further solid state characterization and bioavailability studies in comparison to EPL-NEs and conventional EPL oral tablets in rabbits. 

## 2. Materials and Methods

### 2.1. Materials

EPL and valdecoxib (Sigma-Aldrich, Steinheim, Germany), Avicel PH 102 (FMC Corp., Philadelphia, PA, USA), nanometer-sized amorphous silicon dioxide (SiO2), Triacetin, olive oil, oleic acid, sesame oil, soybean oil, isopropyl myristate (IPM), and isopropyl propionate (IPP) (Sigma-Aldrich, Steinheim, Germany), Transcutol HP, Labrasol, and Labrafil (gift from Gattefosse SAS, Saint-Priest Cedex, France), Pharmaburst 500 (SPI Pharma, Inc. Wilmington, DE, USA), Poly ethylene glycol 400 (PEG 400), tween 40, tween 80, Kolliphor EL, and propylene glycol (PG) (Merck KGaA, Darmstadt, Germany), all other solvents are HPLC grade (Sigma-Aldrich, Steinheim, Germany).

### 2.2. Methodology

#### 2.2.1. Screening of EPL Solubility in NE Components

The solubility of EPL in different lipids (olive oil, oleic acid, sesame oil, soybean oil, IPM, IPP, Labrafil, and Triacetin), surfactants (Labrasol, Tween 40, Tween 80, and Kolliphor EL), and co-surfactants (PEG 400, ethanol, PG, Transcutol HP, and glycerol) was investigated by separately adding excess amounts of EPL to 1 mL of the tested vehicle in small eppendorf. Mixtures were shaken for 72 hours (Hicon, New Delhi, India) at 25 ± 1.0 °C to reach the equilibrium. Samples were centrifuged (REMI Pvt. Ltd., Mumbai, India) at 14,000 rpm for 15 minutes, the supernatant was collected and filtered (0.2 μm membrane), and drug concentration was determined (in triplicate) by a validated HPLC method. Drug solubility was recorded as mg/mL concentrations [2].

#### 2.2.2. Construction of Pseudo-Ternary Phase Diagrams

For construction of the pseudo-ternary phase, the titration method (spontaneous emulsification) was applied as follows: The selected surfactant and co-surfactant were combined in different weight ratios (S_mix_) (coded as: S_1_ (3:1), S_2_ (2:1), S_3_ (1:1), S_4_ (1:2), and S_5_ (1:3)). The selected oil was added to each S_mix_ in different ratios (oil:S_mix_ ratios of 0:100, 5:95, 10:90, 20:80, 30:70, 40:60, 50:50, 60:40, 70:30, 80:20, 90:10, and 100:0) in screw-capped vials and mixed at room temperature until a clear solution was obtained. Phase diagrams were constructed by titrating these samples with aliquots of double-distilled water with continuous stirring for a sufficient amount of time to reach equilibrium. Samples were visually examined for clarity and subsequently classified as being either a clear NE, an emulsion, or gel (if present). The phase behavior of the systems was mapped on triangle phase diagrams with three apices representing S_mix_, oil, and water. The area of clear NE was determined.

#### 2.2.3. Optimization of NE Formulations

After the o/w NE regions in the phase diagrams were identified, 20 plain NE formulations were prepared using different oil:S_mix_:water ratios within the NE region. Plain systems that had a droplet size in the range of 25 to 50 nm, PDI less than 0.3, zeta potential in the range of 25–35 mV, and retained transparency for one week at room temperature were optimized and selected for loading the drug.

#### 2.2.4. Loading of EPL onto NE Formulations

For EPL loading onto NE formulations, an excess amount of drug (150 mg) was added to the optimized oil/S_mix_ mixture and constantly stirred using a mechanical stirrer until a homogenous mixture was obtained. The required amount of water was added in a drop-wise manner with gentle agitation until a clear loaded EPL-NE was produced. 

#### 2.2.5. Characterization of EPL-NE Formulations

##### Drug Loading

To determine the drug loading capacity, EPL-NE formulations were stirred for 24 hours at 25 ± 1.0 °C. Un-dissolved drug was removed by centrifugation at 20,000 rpm for 15 minutes, the supernatant was isolated and filtered (0.2 μm membrane), and the drug content was determined using an HPLC assay method [2]. The analyses were performed in triplicate.

##### Droplet Size, Size Distribution, and Z-Potential Measurement

The average droplet diameter, polydispersity index (PDI), and the droplet surface charge (zeta potential) of the EPL-NE formulations were investigated using photon correlation spectroscopy (Zetasizer 4, Malvern, UK) at 90 °C and 25 °C after dilution with ultra-purified water to an adequate intensity. Each measurement was performed at least in triplicate. The pH value of the samples was maintained at 6.5 ± 0.5.

#### 2.2.6. Loading of Optimized EPL-NE Systems onto Liquisolid Mixtures

##### Calculation of Carrier and Coating Material Amounts

EPL-NE liquisolids were prepared using Avicel PH 102/nano-sized amorphous silicon dioxide as carrier/coat mixtures with a fixed ratio (R) equals 20 that was recommended to give a satisfactory hardness and enhanced release profiles [29]. According to the mathematical model described by Spireas and Bolton [30], the Φ-values of the carrier and the coating materials were calculated for each dry EPL-NE formulation and the calculated L_f_-values (the weight ratio of the liquid medication and carrier powder) were used to determine the amount of carrier, while the amount of coating material was calculated from the applied R-value. These calculated amounts of the carrier and coating materials were predicted to produce formulation mixtures with good flow and compression properties.

##### Preparation of EPL-NE Liquisolids

In a porcelain mortar, dry EPL-NE formulation was manually mixed with carrier material for a suitable amount of time to ensure efficient liquid absorption. The coating material was then added and mixing continued to adsorb excess fluid. This mixing order favors optimal drug release based on a previous publication [31]. Finally, the prepared liquisolid mixtures were directly compressed on a suitable punch-die set (single punch eccentric tablet press EP-1, Erweka, Heusenstamm, Germany) using 10% Pharmaburst 500 SPI (as disintegrant) and 1% magnesium stearate (as a lubricant). Formulations composition is presented in Section 3.4 A batch of 10 EPL-NE loaded liquisolid formulation tablets was prepared.

#### 2.2.7. Evaluation of EP-NE Liquisolids

##### Pre-Compression Evaluation

Flow and compression properties of the prepared EPL-NE loaded liquisolid mixtures were evaluated by measuring the angle of repose (θ), Carr’s index (C_i_), and Hausner ratio [24]. The reported value is an average of three measurements. 

##### Post-Compression Evaluation

The prepared EPL-NE loaded liquisolids were evaluated for weight variation, content uniformity [2], hardness (TBH 325, Erweka, Heusenstamm, Germany), friability (TAR, Erweka, Heusenstamm, Germany), and disintegration time (DST-3/6 automated disintegration tester, Logan, UT, USA) according to standardized United States Pharmacopeia (USP) conditions [32].

#### 2.2.8. In-Vitro Drug Release Studies

Drug release properties of the prepared EPL-NE loaded liquisolids were studied using the USP XXIV dissolution testing apparatus II (UDT-804 paddle dissolution apparatus, Logan, UT, USA) in 500 mL of 0.1 N HCl (pH 1.2) as the dissolution medium at 37 ± 0.5 °C and 50 rpm. At specified time intervals, a 5 mL sample was withdrawn with replacement and the cumulative percentage of drug release was calculated using an HPLC method [2]. For comparison, the drug release rate from corresponding EPL-NE formulations (using dialysis bags with a 12 kDa molecular weight cutoff) and the dissolution rate of unformulated EPL was also determined. The mean of six determinations was recorded.

##### Kinetic Modeling of Release Data

For quantitative kinetic analysis of EPL release profiles from optimized NEs and NE liquisolids, KinetDS 3.0 (Aleksander Mendyk, GNU GPLv3 license, 2007, Kraków, Poland) software was used to investigate the best data fit according to zero order, first order, Higuchi, Korsmeyer-Peppas, and Weibull diffusion models. 

Based on previous in-vitro characterization studies, an EPL-NE liquisolid tablet formulation that showed good flow and compression properties and a high drug release rate were selected and subjected to further solid-state characterization and bioavailability studies.

#### 2.2.9. Solid State Characterizations and Compatibility Studies

Samples of EPL-NE liquisolid formulation, unformulated EPL, and physical mixture were subjected to the following studies described below.

##### Differential Scanning Calorimetry (DSC)

Samples (2–4 mg) were subjected to thermal analysis using a differential scanning calorimeter (Perkin-Elmer DSC4, Shelton, CT, USA.) over a temperature range of (30–300 °C) and a heating rate of 10 °C/minute. DSC thermograms were recorded and analyzed.

##### Infrared Spectroscopy (IR)

Samples were scanned in the range of 4000–500 cm^−1^ at ambient temperature using an IR spectrophotometer (Shimadzu IR-435, Kyoto, Japan), after compression into a transparent disc with KBr 10,000 to 15,000 pounds/inch^2^). IR spectra were recorded and analyzed.

#### 2.2.10. In-Vivo Pharmacokinetic Studies 

The selected EPL-NE loaded liquisolid formulation (treatment A) was compared to the corresponding EPL-NE formulation (treatment B) and conventional oral tablets prepared using the unformulated drug and the same liquisolid formulation excipients (treatment C), in rabbits.

##### Study Design

A single dose bioavailability study with a group of six healthy male albino rabbits (2.5–3.0 kg) was conducted in two phases based on a randomized crossover design. The rabbits were kept on the same diet during the study, with overnight fasting prior to drug administration but free access to water. Rabbits were cannulated through the marginal air vein to collect blood samples. Control plasma samples (2 mL) were withdrawn from each rabbit before drug administration.

A suitable weight of each treatment equivalent to the calculated animal dose based on the Paget and Barnes table [33] was suspended in a minimal volume of water and orally administered to each animal with the aid of gastric gavage. 

Blood samples were collected at pre-determined time intervals for 24 hours following dosing. Blood was collected in heparinized tubes and immediately centrifuged at 3500 rpm for 15 minutes. Plasma was transferred to labeled capped tubes and stored at −20 °C until analysis.

##### Determination of EPL in Plasma

The drug concentration in the collected plasma samples was determined using a sensitive, validated RP-HPLC assay [34], briefly described as follows:

##### Chromatographic Conditions

The mobile phase, consisting of acetonitrile: Water (50:50, v/v), was injected at a flow rate of 1 mL/minute at room temperature in the presence of valdecoxib as an internal standard. A UV detector at λ_max_ 241 nm was used to quantify EPL. 

##### Sample Preparation for Determination of EPL

Frozen plasma samples were thawed at room temperature and were prepared as follows: In 15 mL stoppered test tubes, each plasma sample (1 mL) was added to 50 µL of internal standard solution (10 µg/mL), followed by vortex mixing for one minute. Dichloromethane:diethyl ether [(4:6, v/v), 5 mL] was added and the mixture was shaken for 30 minutes followed by centrifugation at 3000 rpm for 10 minutes. The organic layer was transferred into a new tube and was evaporated to dryness under a nitrogen stream. Finally, the residue was reconstituted in mobile phase (250 µL) and was injected into a chromatographic apparatus. EPL concentration was calculated based on the data obtained from the constructed calibration curve in plasma. 

##### Pharmacokinetic Calculations

Plasma concentration time curves were constructed and the data were manipulated using WinNonlin Professional 4.0.1 software (Pharsight Corp., Cary, NC, USA), followed by calculation of pharmacokinetic parameters (C_max_ (ng/mL), T_max_ (hour), AUC_0–24_ and AUC_0–∞_ (ng·hour/mL, K_el_ (hours^−1^), and t_1/2_ (hours)) for each rabbit.

##### Statistical Analysis of Pharmacokinetic Data

The mean pharmacokinetic parameters were statistically analyzed using the post hoc one-way ANOVA test (Tukey mode) at a p-value > 0.05 and confidence intervals were calculated (IBM-SPSS, Inc., Chicago, IL, USA).

## 3. Results and Discussion

### 3.1. Screening of EPL Solubility in NE Components

**Figure 1:**
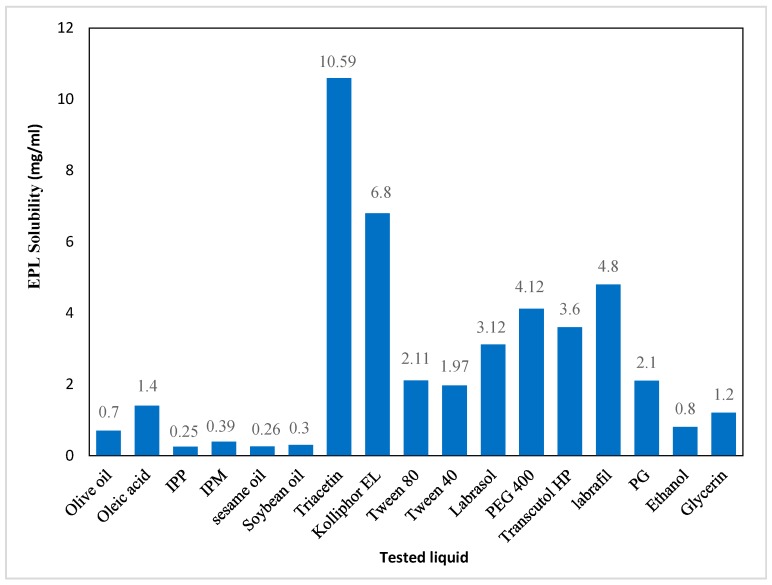
Solubility of EPL in different vehicles.

Selection of appropriate NE formulation components is a significant parameter to obtain a stable system [35]. For hydrophobic drugs, lipid solubility is more important than other factors since it directly affects drug loading and hence the formulation volume for the required dosage. In addition, if drug loading is dependent on adding surfactant and/or co-surfactant, the drug will be prone to precipitation when diluted with GIT due to inhibition of the surfactant/co-surfactant solvation effect [35,36]. Moreover, for oil with low drug solubilizing ability, larger oil amounts may be needed to tolerate the required drug dose. To sequentially attain stable systems, a higher concentration of surfactant is used, which may affect the safety of the NE system [37]. In this work, an oil with maximum drug solubility was selected to prepare NE systems. The solubility of EPL in Triacetin (10.59 mg/mL) was higher than in the other tested oils (Figure 1), which correlated with the medium chain fatty acids and small molar volume of the Triacetin triglyceride that support EPL solubilization [38]. Thus, Triacetin was selected as the oily phase for preparation of NE systems.

The selection of an appropriate SAA for NE formulation depends mainly on its safety, compatibility, and ability to lower the surface free energy at the water/oil interface to produce physically stable systems [36]. Non-ionic SAAs show relatively low physiological toxicity as compared to their ionic equivalents. Non-ionic SAAs also exhibit emulsification properties at a lower concentration, as indicated by a relatively lower critical micelle concentration, which is favorable for oral administration, as surfactants in high concentrations result in GIT irritation. In addition, for o/w NE systems, a hydrophilic nonionic SAA with a higher HLB value (>10) shows a higher positive surface excess that results in accumulation at the oil/water interface, with an efficient decrease in surface tension and improved system stability [35]. In this work, four nonionic SAAs with a high HLB were evaluated for their suitability in the formulation of EPL-NE systems, based on their solubility. Kolliphor EL displayed maximum EPL solubility (6.8 mg/mL) with a high HLB (HLB = 14) (Figure 1), which made it the most suitable SAA for preparing stable EPL-NE systems. 

In order to reduce oil/water interfacial tension significantly and increase interfacial film fluidity, a combination of high HLB surfactants and low HLB co-surfactants is preferable to use of a single SAA. Co-surfactants are usually short or medium chain alcohols (C3–C8) that are added thus that the amount of SAA used can be decreased. Co-surfactants are also distributed between SAA molecules at the oil/water interface that lowers the interaction between polar heads with a subsequent increase in interfacial film flexibility, which efficiently protects the oil droplet and increases hydrocarbon tail oil penetration to form a stable film [39]. Based on drug solubility results, PEG 400 was selected to be used as the co-surfactant for EPL-NE formulations.

Based on the results of EPL solubility presented in Figure 1, Triacetin, Kolliphor EL, and PEG 400 showed maximum drug solubility and were selected as the oil, surfactant, and co-surfactant, respectively, for the preparation of NE formulations.

### 3.2. Pseudo-Ternary Phase Diagram Study

**Figure 2:**
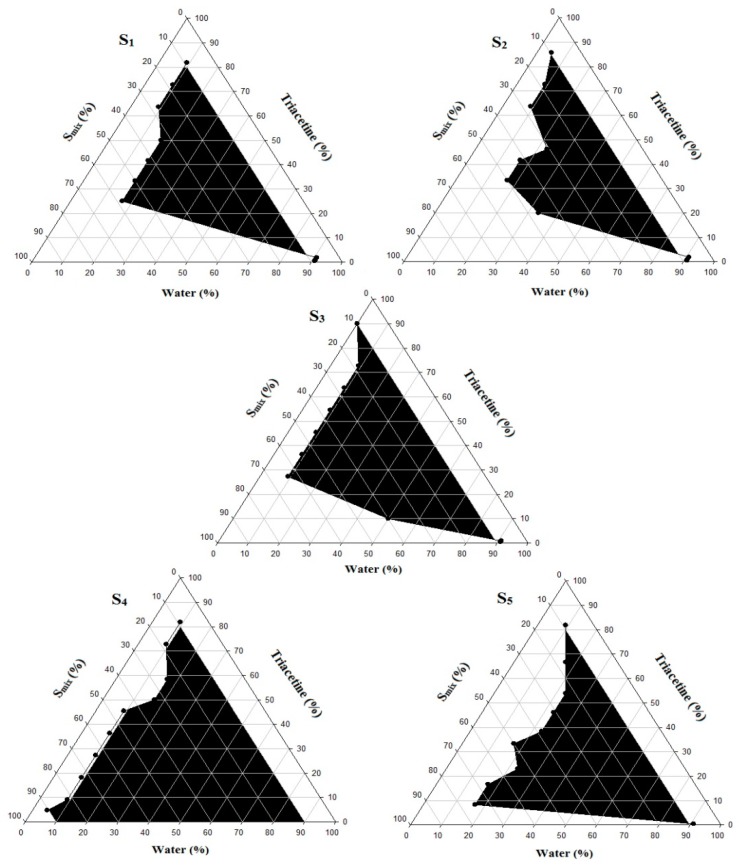
Pseudo-ternary phase diagrams of the quaternary systems containing Triacetin/Kolliphor EL/PEG 400/water at various S_mix_ ratios. **S_1_**: S_mix_ (3:1), **S_2_**: S_mix_ (2:1), **S_3_**: S_mix_ (1:1), **S_4_**: S_mix_ (1:2), **S_5_**: S_mix_ (1:3).

Using Triacetin as oil and Kolliphor EL and PEG 400 as a surfactant/co-surfactant mixture at different ratios (S_mix_), pseudo-ternary phase diagrams were constructed using the water titration method in order to obtain the concentration range of components necessary for preparing the NE. After equilibrium was achieved, each sample was visually checked to determine if clear NE or an emulsion was present. The obtained pseudo-ternary phase diagrams prepared with a different S_mix_ with a field of existence of NE (non-shaded area) are shown in Figure 2. 

Different NE areas were obtained as the S_mix_ changed. The NE area reached its minimum at the highest co-surfactant concentration (Figure 2, S_4_ and S_5_ and a significant increase was observed as the surfactant amount increased (Figure 2, S_1_, S_2_, and S_3_). Thus, an increasing surfactant amount relative to the co-surfactant resulted in significant improvement of the NE area and the maximum was reached at a S_mix_ of 2:1, while further SAA increase (S_mix_ of 3:1) resulted in a narrower NE area. Many studies have shown that the NE area and position are mainly determined by the applied S_mix_ ratio [40] and are affected by the applied oil type and concentration. For a specific oil/surfactant/co-surfactant mixture, the recorded NE area in a phase diagram is an indicator of the emulsification efficiency of the applied S_mix_. In this study, the maximum NE area at S_mix_ of 2:1 was correlated with the maximum emulsification efficacy that resulted in the reduction of the interfacial tension to the minimal level and optimum surfactant/co-surfactant packing with the formation of a highly flexible coherent film at the oil/water interface [36].

Further investigation of the obtained phase diagrams shows that the detected NE region was shifted toward the low-oil apex of the phase diagram, which confirms the importance of selecting an oil of high drug solvation ability to ensure maximum drug loading.

NE formulations with a high SAA concentration are unfavorable since this may result in in vivo irritation and also decreases the thermodynamic activity of the drug in the vehicle, which may affect release of the drug from the applied dosage form [41]. Therefore, NE formulation systems capable of attaining enhanced entropy and significantly lower oil/water interfacial free energy with the minimum S_mix_ were considered to be thermodynamically stable and were selected for further study.

### 3.3. Optimization of EPL-NE

Based on the constructed pseudo-ternary phase diagram, 20 plain NE formulations were prepared and evaluated for droplet size, zeta potential, and PDI (data not shown). Only five plain NE systems met the specified optimization criteria of droplet size in the range of 25 to 50 nm, zeta potential ranging from −25 to −35 mV, a PDI less than 0.3, and extended transparency for one week at room temperature. The composition of these NE formulations was as follows: At S_mix_ of 3:1, Triacetin (10%), Kolliphor EL (30%), PEG 400 (7%), and water (53%); and Triacetin (15%), Kolliphor EL (33%), PEG 400 (11%), and water (41%). At S_mix_ of 2:1; Triacetin (17%), Kolliphor EL (30%), PEG 400 (14%), and water (39%); and Triacetin (24%), Kolliphor EL (30%), PEG 400 (17%), and water (28%). At S_mix_ of 1:3, Triacetin (5%), Kolliphor EL (12%), PEG 400 (36%), and water (47%). It was noted that higher oil concentrations were obtained for NE systems prepared at S_mix_ of 2:1, as indicated by maximum emulsification efficacy. The optimized NE systems were selected for drug loading.

### 3.4. Characterization of EPL-NE Formulations

#### 3.4.1. Drug Loading

The drug loading capacity of the prepared NE formulations varied from 37.91% (F5) to 99.11% (F4) (Table 1), depending on oil content. The NE formulation with a higher oil content contained higher amounts of the entrapped drug, which correlated with the higher solubility of the drug in Triacetin (oil) and also confirms the importance of drug solubility as the main criterion for selection of oil. 

#### 3.4.2. Particle Size and Size Distribution 

All prepared EP-NEs formulations had acceptable droplet sizes, ranging from 32.81 nm to 48.41 nm, with a narrow PDI, in the range of 0.160 to 0.240 (Table 1). PDI correlates the standard deviation of the droplet size with the calculated mean size, which is a criterion for the uniformity of size distribution within the NE formulation [20]. The small PDI values indicate that the systems are mono-dispersed, have a narrow particle size distribution, and have a higher degree of stability [42]. Further investigation of the results showed that the oil content had a significant positive effect on droplet size, which could be explained by the increased viscosity of the mixture due to increased drug concentration. Surfactant and/or S_mix_ did not have an effect on droplet size, mainly due to the proximity of the applied SAA concentration in the optimized systems.

#### 3.4.3. Zeta Potential

Zeta potential describes the electro-kinetic potential of colloidal systems and its value reflects the intensity of electric charges at the oil/water interface. Zeta potential is the main parameter that determines the physical stability of the dispersion, where repulsion between similar charges acts against the Oswald ripening and prevents droplet aggregation with subsequent phase separation [43]. Zeta potential is controlled by the type and concentration of the applied SAA, system composition, drug characteristics, vehicle nature, and presence of electrolytes. Generally, for the specific NE, it was previously claimed that a zeta potential of ±30 mV indicates sufficient physical stability of a system [20,44].

All optimized EPL-NE formulations had a zeta potential in the range of −25.2 to −35.7 mV (Table 1), indicating good stability of the optimized systems.

The SAA affects zeta potential either directly through neutralization of surface charges or indirectly through its effect on the particle size and surface area of the internal phase droplets [45]. In this study, the applied SAA and co-surfactant are nonionic and had a negligible effect on surface charge; additionally, the proximity of SAA concentration in all optimized formulations downplays its effect on zeta potential. The negative charge of the droplet surface mainly correlated with the fatty acid content of the lipoid phase, with a slight difference between the NE systems according to their lipid content and droplet size [19].

### 3.5. EPL-NE Liquisolids

The liquisolid technique is an advanced approach used mainly for enhancing the water solubility of a drug by mixing it with a suitable water miscible vehicle and loading onto a powder mixture of high absorbent and/or adsorbent properties (carrier and coat) in specified weights calculated according to the proposed mathematical model to achieve complete dryness and ensure good flow and compressibility characteristics [30]. In other words, liquisolid compacts could be described as liquid medications with an adequate flow that are compressed into a powdered form. In this work, a liquisolid mixture was applied as a carrier for the prepared EPL-NE formulations with good flow characteristics and that was suitable for direct compression using Avicel/nano-sized silicone dioxide as a carrier/coat mixture; thus, no water miscible vehicle was added and the calculations were dependent on the weight of the dry EPL-NE formulation that was loaded as a liquid medication. 

According to the mathematical model, the flowable liquid retention potentials (Ф-Value) of Avicel and nano-sized silicone dioxide (R = 20) were used to calculate the liquid load factor (L_f_); the latter was used to calculate the amounts of carrier and coat materials that depended on the weight of the applied dry EPL-NE formulation according to the following equation:

L_f_ = W/Q (1)

where W is the weight of the dry EPL-NE formulation and Q is the weight of the carrier. The amount of coating material was calculated from the R-value. The composition and flow characterization of the prepared EPL-NE liquisolids are shown in Table 2.

Highly flowable characteristics are essential for mixtures that are prepared for direct compression to guarantee homogenous mixing, efficient die fill, uniform tablet weight, precise drug content, and ultimately accepted therapeutic value [15]. The flow characteristics of the prepared EPL-NE liquisolids were assayed by measuring the angle of repose, Carr’s index, and Hausner ratio. 

Based on the results in Table 2, all prepared EPL-NE liquisolid mixtures showed very good flow characteristics, with an angle of repose in the range of 27.61° to 32.41°, Carr’s index values less than 20 (in the range of 16.02 to 19.37%), and the Hausner ratio close to unity [24]. These results designate the precision of the applied calculations of different model parameters and indicate the suitability of selecting a liquisolid technique as a carrier for the prepared EPL-NE formulation for direct compression. 

### 3.6. Characterization of EPL-NE Liquisolid Tablets

Characterization results of the prepared EPL-NE liquisolid tablets are shown in Table 3. All formulations showed a small weight variation and high drug content, ranging from 96.12% to 100.15%, which, according to standardized pharmacopeial limits, correlates with and indicates that the prepared liquisolid formulation mixtures had good flow characteristics. Hardness values above 50 N and friability less than 1% indicate good mechanical properties and adequate breaking strength, which can be correlated with the high compressibility characteristics of the formulation components (Avicel and Pharmaburst). Selection of Pharmaburst-500 SPI for use as a superdisintegrant for liquisolid tablet formulations not only enhanced the mechanical properties but also had a positive effect on disintegration. Pharmaburst is a co-processed mixture of mannitol, starch, crosspovidone, crosscarmellose-Na, and silica [46]. The combined wicking and swelling properties of both carmellose and povidone resulted in rapid tablet disintegration within 41 to 53 seconds for formulations F1 and F4, respectively.

### 3.7. Drug Release Study

**Figure 3:**
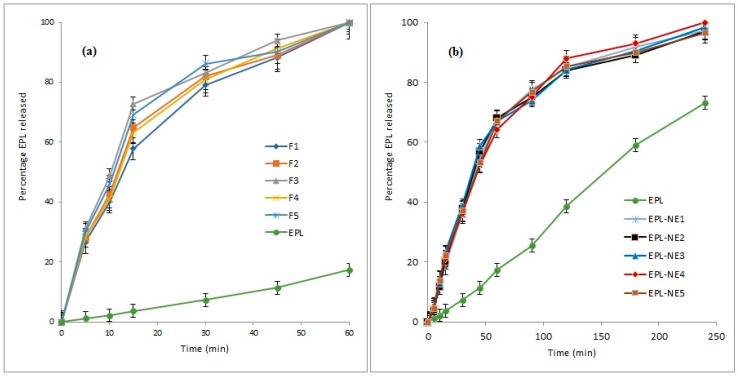
Release profiles of EPL from (**a**): NE-Liquisolids and (**b**): NE formulations in comparison to unformulated EPL.

Figure 3 shows the dissolution profiles of EPL from different prepared EPL-NE liquisolid tablet formulations in comparison to EPL-NEs and unformulated EPL in 0.1 N HCl (pH 1.2). The percent drug release showed a remarkable increase from all liquisolid formulations, in which approximately 90% of the drug was released after 45 minutes and drug release was complete within one hour, as compared to 39.6% and 47.3% for the unformulated drug after 45 and 60 minutes, respectively. For EPL-NE, a significant improvement of drug release was observed but with a sustained pattern, in which nearly complete drug release (greater than 97%) occurred within four hours. Further investigation of the release data indicated that the drug release profiles of both prepared EPL-NEs and EPL-NE liquisolids showed a biphasic release pattern, with rapid initial drug release in which 60–70% of the drug dose was released within one hour and 15 minutes for EPL-NEs and EPL-NE liquisolids, respectively. This could be explained by the higher wettability of the drug in contact with water due to the SAA effect that is favored in liquisolids by the presence of the finely-sized particle component of liquisolid (nano-silica) that acts as a distributing agent via the adsorption effect on a larger surface area and rapid drug partitioning into diluted dissolution medium, mainly from small droplets. A slower drug release pattern in the second phase could be correlated with the decline in oil/water drug partitioning from larger droplets under the effect of increasing media drug concentration according to the Noyes-Whitney equation [47]. The effect of droplet size distribution (PDI) on drug release from nanoemulsion systems was verified by Ritger and Peppas [48] and confirmed by many researchers [49]. Our results are in agreement with many studies that concluded that conventional NE formulations usually result in enhancement of hydrophobic drug dissolution but with a sustained release pattern under the effect of oil and interfacial film barriers [50]. However, in this work, it was clearly seen that the loading of the NE formulation onto a liquisolid mixture resulted in significant enhancement of both the amount released and the rate. This could be correlated to the fact that even though the drug prepared in a solid dosage form, it is detained within the formulation powder blend in solution or in an almost molecularly dispersed form, which causes a significant increase in surface area that is available for dissolution and the degree of subdivision of the drug loaded oil droplets with improved wetting characteristics [25]. This effect on the drug release profile is highly advantageous since it allows faster drug absorption to therapeutic levels in addition to the overall increase in drug absorption, which mainly occurs in gastric and upper duodenal segments [12,14]. 

EPL release data from the EPL-NEs and EPL-NE liquisolids was fitted to different kinetic models and then the best fit was selected based on the calculated correlation coefficient (R^2^) value for each model. The Weibull model showed the best fit with an R^2^ value that exceeded 0.9994 for the EPL-NE. For EPL-NE liquisolids, drug release followed the Korsemeyer-Peppas model with R^2^ values greater than 0.9986. These results indicate uniformity of drug solubility and distribution within the lipid phase, with a subsequent complex mixed dissolution-diffusion release pattern that is predictable in NE systems and typically described by the Weibull model. For the Korsemeyer-Peppas model, a number of synchronized steps usually occur that are expected for modified tablets dosage forms including diffusion of water, tablet swelling with subsequent drug diffusion, and dissolution. These results clearly demonstrate the effect of liquisolid loading on drug release from EPL-NE formulations [51].

Based on previous in vitro characterization results, EPL-NE liquisolid formulation No. 4 (F4) was selected and subjected to further solid-state characterizations and bioavailability studies.

### 3.8. Solid State Characterizations and Compatibility Studies

Solid state characterizations and compatibility studies were performed with the optimized EPL-NE liquisolid formulation (F4) in comparison to formulation physical mixture and unformulated drug.

**Figure 4:**
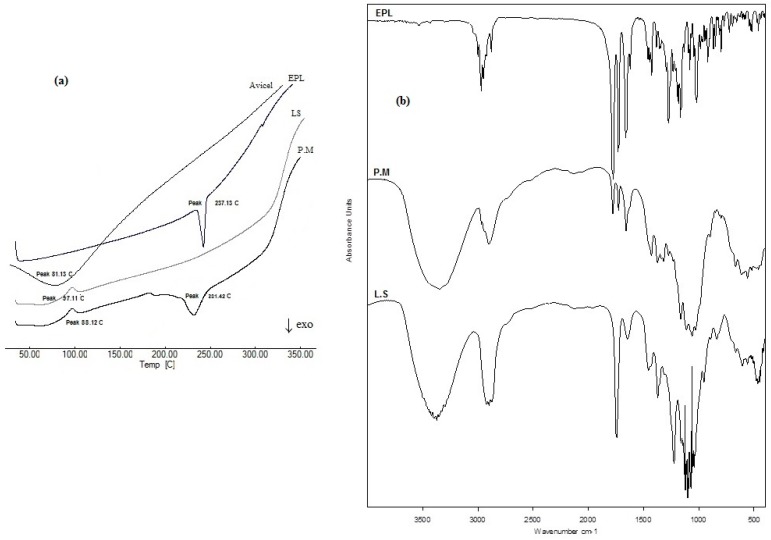
Solid state characterization of EPL-NE liquisolid formulation using (**a**) differential scanning calorimetry (DSC) and (**b**) FTIR.

DSC is a reliable and simple tool to test for material purity, formulation mixture compatibility, and describing drug crystalline properties in a specific blend, which is affected by drug solubility and/or distribution state within the mixture. Figure 4a shows the DSC thermogram of unformulated EPL with a sharp endothermic melting peak at 231.13°C in accordance with previously published data [12], indicating drug purity, which completely disappeared in thermograms of the liquisolid formulation under the effect of drug solubility within the oil core and loss of crystallinity. The drug melting peak was retained in the thermogram of the physical mixture without the appearance of any new peaks or other thermal events, indicating compatibility between formulation mixture components. Figure 4b shows the IR spectrum of unformulated EPL with the main characteristic functional groups at 2988.65 cm^−1^ (C–H stretching), 1778.08 cm^−1^ (anhydride O–C=O stretching), 1726.22 cm^−1^ (C=O ester stretching), and (1657.64 cm^−1^) C=O stretching. IR spectra of EPL-NE liquisolid and the physical mixture showed no chemical changes where all drug functional groups were retained, which confirmed the compatibility of EPL with formulation additives.

### 3.9. In Vivo Characterization Studies

**Figure 5:**
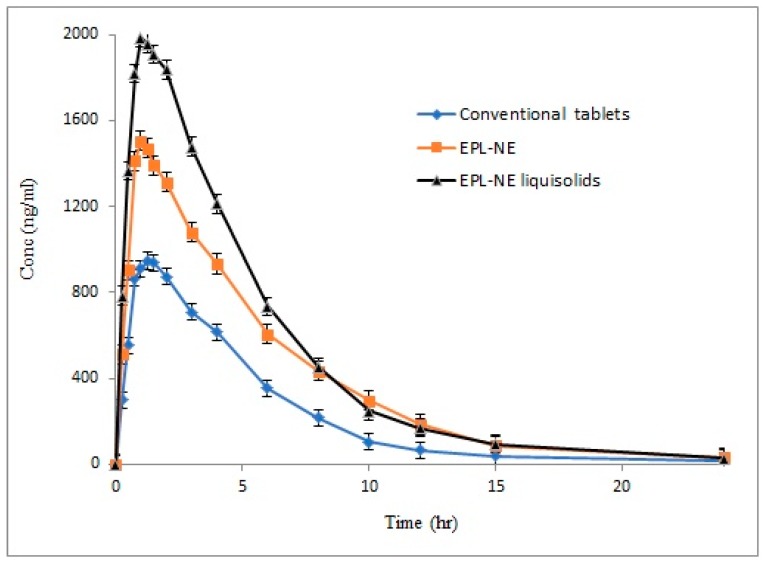
Mean plasma concentration time curves of EPL from different tested forms after administration to rabbits.

To study the effect of increased solubility and the improved release rate of EPL from the prepared NE liquisolid formulation on drug absorption and bioavailability, the optimized formulation (F4) was compared to the corresponding EPL-NE and conventional oral tablets in rabbit bioavailability studies. The mean pharmacokinetic parameters of EPL of different tested formulations are summarized in Table 4 and the mean plasma concentration-time profiles are illustrated in Figure 5.

Bioavailability is generally defined as the rate and extent of drug absorption. In bioavailability calculations of plasma data, the extent of absorption is usually quantified and described by both area under plasma concentration time curve (AUC) and the drug maximum plasma concentration (C_max_) while time elapsed to reach maximum plasma concentration (T_max_) is the parameter that describes absorption rate [15]. In this study, major changes in all pharmacokinetic parameters were observed, indicating an improvement in both the rate and extent of drug absorption. For liquisolid formulations, the mean C_max_ and AUC_0–α_ were increased and reached 1986.76 ng/mL and 11,328.11 ng.hr/mL, respectively, as compared to 1505.51 ng/mL and 9292.19 ng.hr/mL for the EPL-NE formulation, while lower plasma concentrations were observed for conventional oral tablets, with C_max_ and AUC_0–α_ values of 946.34 ng/mL and 5383.56 ng·hr/mL, respectively. The results also showed a shortened T_max_ of 0.95 hours and 1.04 hours for liquisolid and NE formulations, respectively, as compared to 1.38 hours for conventional tablet formulation. As dissolution is the rate determining step in the absorption process of BCS class II drugs, these results correlate with the significant increase in drug dissolution from liquisolid formulations, the positive effect of NE on permeation through the GIT lumen, and para-cellular absorption that allows the drug to escape liver metabolism, with subsequent higher plasma levels.

Statistical analysis of the calculated pharmacokinetic parameters is shown in Table 5 and Table 6, with 95% confidence intervals. According to these results, a significant difference was observed at the selected probability level. Additional data analysis was performed using the post-hoc tukey model to pinpoint the exact position of the observed difference, which indicated a significant difference in both the rate and extent of drug absorption for EPL-NE liquisolid as compared to conventional tablets but the only significant difference in the extent of absorption that was observed was in comparison with the EPL-NE formulation. A significant difference in both the rate and extent of drug absorption between conventional tablets and EPL-NE formulation was observed. The 95% confidence intervals had narrow ranges and excluded zero, which indicates data accuracy, the strength of the results, and confirmed the rejection of the null hypothesis.

## 4. Conclusions

NEs are lipid formulations that can easily pass through the GIT membranes and bypass liver degradation due to lymphatic absorption, which significantly enhances the bioavailability of poorly soluble drugs, especially those that suffer liver metabolism. In this work, EPL as a BCS class II drug model that normally suffers extensive liver degradation was formulated into an NE using Triacetin (oil) and Kolliphor EL/PEG 400 (S_mix_) in different ratios. Optimized NE formulations were loaded onto free flowing, highly compressible liquisolid tablet mixtures for oral delivery and the drug bioavailability of these NE formulations were compared to conventional oral tablets and NE formulation. Drug release from optimized EPL-NE liquisolids was significantly increased, with a high rate that exceeded 90% within 45 minutes, while EPL-NE also showed significant drug release but with a sustained pattern over four hours. The rate and extent of drug absorption were significantly increased from liquisolids as compared to NE formulations and conventional tablets, as indicated by the AUC, C_max_, and T_max_, in which bioavailability from liquisolids reached 2.1 relative to conventional tablets and 1.21 relative to the NE formulation. The bioavailability of EPL from NE formulation was increased by 1.72 relative to conventional oral tablets.

These results support the conclusion that the EPL-NE liquisolid mixture is a promising dosage form for oral delivery with good flow and compression characteristics that increase the release rate, enhance drug absorption, and allows the drug to escape liver degradation, with a subsequent significant increase in drug bioavailability. In addition, Nanoemulsion Liquisolids, are expected to be a promising oral delivery system for BCS class II drugs with poor bioavailability especially those liable to extensive liver degradation. Further studies on other drug models in terms of pK, logP, and permeability are required to confirm the suitability of nanoemulsion liquisolids as an oral delivery system to increase drug bioavailability and protect from liver degradation.

## Figures and Tables

**Table 1:** Characterization of the prepared EPL- nanoemulsions (NE) formulations.

Code	S_mix_	Loading Capacity (%)	Droplet Size (nm)	Zeta Potential	PDI
F1	S_1_	67.42 ± 1.23	37.63	−27.3	0.196 ± 0.013
F2		86.74 ± 1.46	42.99	−31.4	0.240 ± 0.053
F3	S_2_	95.31 ± 1.91	43.74	−29.8	0.181 ± 0.099
F4		99.11 ± 1.11	48.41	−35.7	0.160 ± 0.014
F5	S_5_	37.91 ± 1.09	32.81	−25.2	0.230 ± 0.017

PDI: Polydispersity index.

**Table 2:** Composition and pre-compression characterization of EPL-NE liquisolid formulations.

F	EPL (mg)	L_f_	Q	q	Disg.	W (mg)	θ	Ci%	Hausner Ratio
F1	25	0.21	223.8	11.9	23.6	291.7	28.75	16.92	1.17
F2			280.9	14.1	29.5	352.4	31.82	18.11	1.16
F3			290.5	14.5	30.5	363.5	32.41	19.37	1.13
F4			338.1	16.9	35.5	419.1	30.82	17.41	1.15
F5			252.4	12.6	26.5	319.2	27.61	16.02	1.07

F: Formulation. L_f_: Liquid load factor. Q: Carrier weight. q: Coat weight. θ: Angle of repose. Ci%: Carr’s index. Disg.: Disintegrant weight. W: Unit dose weight.

**Table 3:** Post-compression characterization of EPL-NE liquisolid tablets.

F	Average Weight (mg) ± S.D.	Drug Content (%) ± S.D.	Thickness (cm)	Diameter (cm)	Friability (%)	Hardness (N)	Dis. Time (Seconds)
F1	289.12 ± 3.6	97.89 ± 2.1	0.15	0.8	0.5	55	41
F2	349.7 ± 2.9	99.19 ± 1.3	0.21	0.8	0.4	56	45
F3	365.2 ± 3.1	100.15 ± 0.8	0.21	0.8	0.4	59	47
F4	422.1 ± 3.2	100.08 ± 0.2	0.23	0.8	0.3	63	53
F5	317.9 ± 2.9	96.12 ± 3.4	0.18	0.8	0.6	55	44

N: Newton. Dis.: Disintegration.

**Table 4:** Mean pharmacokinetic parameters of EPL after administration to rabbits in different forms.

Pharmacokinetic Parameter	EPL-NE Liquisolids	EPL-NE	EPL Tablets
C_max_ (ng/mL)	1986.76 ± 53.6	1505.51 ± 24.91	946.34 ± 46.27
T_max_ (hr)	0.95 ± 0.048	1.04 ± 0.023	1.38 ± 0.021
K (hr^−1^)	0.187 ± 0.031	0.164 ± 0.042	0.196 ± 0.028
t_1/2_ (hr)	3.719 ± 0.12	4.238 ± 0.18	3.547 ± 0.07
AUC _(0–4)_ (ng·hr/mL)	11178.56 ± 74.47	9108.76 ± 35.27	5293.15 ± 27.33
AUC _(0–α)_ (ng·hr/mL)	11328.11 ± 35.78	9292.19 ± 23.38	5383.56 ± 18.72

**Table 5:** One way ANOVA test for EPL pharmacokinetic data from different forms in rabbits.

Parameter	Source	Sum of Squares	df	Mean Square	F	Sig.
C_max_ (ng/mL)	Between Groups	3,131,899.76	2	1,565,949.88	4584.19	<0.001
	Within Groups	5123.96	15	341.597		
	Total	3,137,023.72	17			
T_max_ (hr)	Between Groups	0.444	2	0.222	16.84	
	Within Groups	0.198	15	0.013		
	Total	0.642	17			
AUC _(0–24)_ (ng·hr/mL)	Between Groups	106,305,369.09	2	53,152,684.55	285.95	
	Within Groups	2,788,214.49	15	185,880.97		
	Total	109,093,583.59	17			
AUC _(0–α)_ (ng·hr/mL)	Between Groups	108,822,905.28	2	54,411,452.64	255.43	
	Within Groups	3,195,313.44	15	213,020.89		
	Total	112,018,218.72	17			
K (hr^−1^)	Between Groups	0.001	2	0.001	9.38	0.002
	Within Groups	0.001	15	0.000		
	Total	0.003	17			
t_1/2_ (hr)	Between Groups	0.804	2	0.402	8.88	0.003
	Within Groups	0.679	15	0.045		
	Total	1.482	17			

df: degree of freedom.

**Table 6:** Post-Hoc statistical analysis of the calculated means with 95% confidence intervals.

Parameter	Group		Mean Difference	Confidence Interval	
				Lower	Upper
C_max_ (ng/mL)	EPL-NE Liquisolids	EPL Tablets	1020.468 *	992.751	1048.185
		EPL-NE	465.968 *	438.251	493.685
T_max_ (hr)		EPL Tablets	−0.334 *	−0.506	−0.161
		EPL-NE	0.0011	0.0007	0.012
K (hr^−1^)		EPL Tablets	0.0059	0.003	0.019
		EPL-NE	0.0214 *	0.008	0.035
t_1/2_ (hr)		EPL Tablets	−0.129	−0.4481	−0.120
		EPL-NE	−0.499 *	−0.8176	−0.179
AUC _(0–24)_ (ng·hr/mL)		EPL Tablets	5864.821 *	5218.263	6511.379
		EPL-NE	2049.661 *	1403.103	2696.218
AUC _(0–α)_ (ng·hr/mL)		EPL Tablets	5924.305 *	5232.155	6616.457
		EPL-NE	2022.642 *	1330.491	2714.793

*: Significant model term.
